# Dataset on antixenosis and antibiosis of chili fruit by fruit fly (*Bactrocera dorsalis*) infestation

**DOI:** 10.1016/j.dib.2019.103758

**Published:** 2019-03-07

**Authors:** Tati Suryati Syamsudin, Ahmad Faizal, Rinda Kirana

**Affiliations:** a School of Life Sciences and Technology, Institut Teknologi Bandung, Ganesa 10 Bandung 40132, Indonesia; b Indonesian Vegetable Research Institute (IVEGRI), Tangkuban Parahu 517 Bandung Barat 40391, Indonesia

**Keywords:** Choice, Fitness index, No-choice, Oviposition puncture

## Abstract

This article contains the data on chili antixenosis and antibiosis to fruit fly (*Bactrocera dorsalis*) infestations. The data was collected from the experiment. Fifty chili varieties (*Capsicum* spp.) were planted in the screen house and subjected to antixenosis and antibiosis tests. The antixenosis test was evaluated using choice and no-choice methods. The data observed was the number of oviposition punctures by fruit fly on the chili fruits. The antibiosis test was conducted on chili fruits using the Fitness Index method. The data observed were the percentage of pupa (%), the weight of pupa (mg), duration of larva-pupa (day), and duration of pupa-imago (day).

**Table:** Specifications table

Subject area	Agriculture and Biological Science
More specific subject area	Entomology
Type of data	Table
How data was acquired	Laboratory observations
Data format	Raw and Analyzed
Experimental factors	Fifty varieties of chili fruit were infested by female fruit fly imagoes
Experimental features	The antixenosis test was evaluated using choice and no-choice methods. The data observed was the number of oviposition punctures by fruit fly on the chili fruits surface. The antibiosis test was conducted on chili fruits using the Fitness Index method. The data observed were the percentage of pupa (%), the weight of pupa (mg), duration of larva-pupa (day), and duration of pupa-imago (day).
Data source location	Lembang, West Java, Indonesia
Data accessibility	The data are available with this article
Related research article	Most relevant research article are: (1) M. Aluja, J. Arredondo, F. Díaz-Fleischer, A. Birke A, J. Rull, J. Niogret, N. Epsky, Susceptibility of 15 mango (Sapindales: Anacardiaceae) cultivars to the attack by *Anastrepha ludens* and *Anastrepha obliqua* (Diptera: Tephritidae) and the role of underdeveloped fruit as pest reservoirs: management implications, J. Econ. Entomol. 107 (2014) 375–388. https://doi.org/10.1603/EC13045. (2) Y. Hidayat, N. Heather, E. Hassan, Repellency and oviposition deterrence effects of plant essential and vegetable oils against female Queensland fruit fly *Bactrocera tryoni* (Froggatt) (Diptera: Tephritidae), Aust. J. Entomol. 52 (2013) 379–386. https://doi.org/10.1111/aen.12040.

**Table:** 

**Value of the data** • This data informed the response of fifty variety of chili fruit to fruit fly (Bactrocera dorsalis) infestation. • The data could be used by other researchers on fruit fly preference with a different variety of chili fruits. • This data could support breeding programs for a selection of chili fruit variety to fruit fly infestation.

## 1 Data

We present the data of antixenosis and antibiosis of fruit fly (*Bactrocera dorsalis*) infestation on fifty chili varieties based on response of oviposit female fruit fly on chili fruits (Table 1). The Data was generated from antixenosis test conducted with choice and no-choice methods. The observed parameter for measuring the level of antixenosis was the number of oviposition punctures by female fruit fly on the fruit surface. Oviposition deterrent index of choice method was presented in Supplementary 1. While the no-choice method was presented in Supplementary 2. An antibiosis test conducted by inserting fruit fly eggs on chili fruits. The data observed were the percentage of pupa (%), the weight of pupa (mg), duration of larva-pupa (day), and duration of pupa-imago (day). The data obtained provided supplementary data for this article (Supplementary 3).

**Table 1:** Antixenosis and Antibiosis 50 varieties of chili fruits by fruit fly (*Bactrocera dorsalis*) infestation.

Chili Variety	Antixenosis-Deterrent Index		Antibiosis (Fitness Index)
	Choice	No-choice	
1	76.79 ± 5.17	75.93 ± 2.45	0.05 ± 0.02
2	92.59 ± 3.70	80.56 ± 12.73	0.10 ± 0.05
3	100.00 ± 0.00	80.56 ± 10.52	0.13 ± 0.07
4	79.26 ± 5.79	46.30 ± 8.07	0.50 ± 0.25
5	93.09 ± 4.81	94.44 ± 1.60	0.41 ± 0.20
6	46.01 ± 8.89	11.11 ± 8.49	2.17 ± 1.08
7	100.00 ± 0.00	94.44 ± 4.24	0.20 ± 0.10
8	100.00 ± 0.00	87.04 ± 9.12	1.78 ± 0.89
9	100.00 ± 0.00	83.33 ± 5.78	0.36 ± 0.18
10	92.59 ± 3.70	74.07 ± 8.23	0.21 ± 0.11
11	100.00 ± 0.00	92.59 ± 4.63	0.09 ± 0.04
12	92.59 ± 3.70	93.52 ± 6.48	1.92 ± 0.96
13	95.06 ± 3.27	82.41 ± 6.68	0.69 ± 0.35
14	100.00 ± 0.00	84.26 ± 7.91	1.28 ± 0.64
15	100.00 ± 0.00	90.74 ± 2.45	0.79 ± 0.40
16	92.59 ± 3.70	70.37 ± 4.63	0.15 ± 0.07
17	82.09 ± 6.63	87.04 ± 8.07	1.60 ± 0.80
18	92.59 ± 3.70	93.52 ± 5.16	0.08 ± 0.04
19	76.79 ± 5.17	64.81 ± 0.93	0.33 ± 0.17
20	97.53 ± 2.47	68.52 ± 7.41	2.66 ± 1.33
21	95.06 ± 3.27	92.59 ± 7.41	2.23 ± 1.12
22	100.00 ± 0.00	95.37 ± 2.45	0.64 ± 0.32
23	100.00 ± 0.00	90.74 ± 5.16	0.42 ± 0.21
24	57.76 ± 4.89	55.56 ± 2.78	1.05 ± 0.53
25	71.09 ± 5.89	87.04 ± 9.12	0.25 ± 0.12
26	73.20 ± 6.23	37.04 ± 7.91	2.24 ± 1.12
27	82.09 ± 6.63	58.33 ± 2.78	0.97 ± 0.49
28	74.32 ± 4.31	63.89 ± 12.11	2.62 ± 1.31
29	78.77 ± 7.97	61.11 ± 11.23	1.83 ± 0.92
30	61.82 ± 5.63	46.30 ± 6.07	4.42 ± 2.21
31	66.13 ± 6.60	57.41 ± 10.19	4.11 ± 2.05
32	63.05 ± 5.02	27.78 ± 5.56	2.11 ± 1.05
33	78.77 ± 4.73	66.67 ± 13.98	4.69 ± 2.35
34	83.70 ± 5.64	89.81 ± 4.90	0.98 ± 0.49
35	74.05 ± 7.53	54.63 ± 10.31	0.91 ± 0.46
36	63.95 ± 6.30	53.70 ± 3.70	1.01 ± 0.50
37	88.15 ± 5.02	58.33 ± 15.30	0.47 ± 0.24
38	100.00 ± 0.00	78.70 ± 9.67	0.38 ± 0.19
39	95.06 ± 3.27	85.19 ± 0.93	1.12 ± 0.56
40	90.62 ± 4.99	88.89 ± 8.33	2.05 ± 1.03
41	68.26 ± 4.04	71.30 ± 2.45	1.46 ± 0.73
42	47.82 ± 3.88	50.00 ± 10.02	0.65 ± 0.33
43	65.93 ± 2.96	23.15 ± 8.07	0.86 ± 0.43
44	50.70 ± 6.19	39.81 ± 9.26	2.44 ± 1.22
45	100.00 ± 0.00	99.07 ± 0.93	0.16 ± 0.08
46	60.93 ± 9.53	50.93 ± 5.16	3.23 ± 1.62
47	87.65 ± 3.90	79.63 ± 10.19	0.06 ± 0.03
48	78.27 ± 3.35	75.00 ± 13.70	0.02 ± 0.01
49	78.27 ± 3.35	54.63 ± 16.38	0.45 ± 0.22
50	48.41 ± 7.39	22.22 ± 8.49	3.15 ± 1.58

## 2 Experimental design, materials, and methods

The antixenosis response of chili to fruit fly (*Bactrocera dorsalis*) infestation (oviposition) was conducted based on the choice and no-choice preference methods developed by Aluja et al. [1] with some modifications. The choice test was conducted in a cage (40 cm × 40 cm x 40 cm). A total of 450 chili fruits from 50 different varieties were used. Fifty chili fruits were placed into a cage and infested by 25 pairs of fruit flies for 48 hours. This experiment was repeated nine times. The no-choice test was conducted using 750 chili fruits from 50 different varieties. Fifty cages (20 cm × 20 cm x 20 cm) were used for this test. Each cage contained five chilies representing each variety and was exposed to 25 pairs of fruit fly for 48 hours. These experiments were replicated three times. Fruit fly infestation was indicated by the presence of a small puncture on the fruit surface. The preference was calculated based on oviposition punctures. In the choice method, the antixenosis level against fruit fly infestation was calculated using an oviposition deterrent index based on Simmonds et al. [2] and Hidayat et al. [3]. The level of antixenosis to fruit fly infestation of the no-choice method was calculated based on Bentley et al. [4] and Hidayat et al. [3].

An antibiosis experiment was set up according to Hennessey et al. [5] with some modifications. Female fruit fly imagoes could lay eggs in an artificial breeding tube. The eggs were then inserted into chili fruits. The number of inserted eggs was determined by the proportion of the fruit's weight (three eggs per gram of fruit). The infested chili fruits were laid on the plastic glass (8.2 cm in diameter) containing sterilized sawdust and incubated in a controlled culture chamber (28±2 °C temperature, 75% ± 10% humidity). The presence of pupa and imago was observed every day. The number and weight of pupa were recorded until 15 days after egg infestation. The data observed were the percentage of pupa (%), the weight of pupa (mg), duration of larva-pupa (day), and duration of pupa-imago (day). After that, the pupae were moved and laid in a new plastic glass until emergence. The imagoes were counted every day until 15 days. The level of antibiosis of chili to fruit fly infestation was calculated using the Fitness Index (FI) based on Jallow and Zalucki [6] and Balagawi et al. [7].
