# Supplementary material for: Dataset on antixenosis and antibiosis of chili fruit by fruit fly (Bactrocera dorsalis) infestation
Source: Data Brief. 2019 Mar 7;23:103758. doi: 10.1016/j.dib.2019.103758 (PMC6660636; doi:10.1016/j.dib.2019.103758)
Supplement: Multimedia component 1 [file mmc1.docx]

DECLARATION

We are the authors of the title of manuscript” Dataset on antixenosis and antibiosis of chili fruit by fruit fly (*Bactrocera dorsalis*) infestation” declared there is no conflict of interest

Tati Suryati Syamsudin

Ahmad Faizal

Rinda Kirana
